# Concurrent Experience of Self-Reported Mental Health Symptoms and Problematic Substance Use During the First Two Years of the COVID-19 Pandemic Among Canadian Adults: Evidence from a Repeated Nationwide Cross-Sectional Survey

**DOI:** 10.3390/ijerph21121644

**Published:** 2024-12-10

**Authors:** Md Sabbir Ahmed, Mary Bartram, Robert Gabrys, Mansfield Mela, Nazeem Muhajarine

**Affiliations:** 1 Saskatchewan Population Health and Evaluation Research Unit, University of Saskatchewan, 104 Clinic Place, Saskatoon, SK S7N 2Z4, Canada; sabbir.ahmed@usask.ca; 2 Department of Community Health and Epidemiology, College of Medicine, University of Saskatchewan, Saskatoon, SK S7N 5E5, Canada; 3 Mental Health Commission of Canada, Ottawa, ON K1R 1A4, Canada; mary.bartram@carleton.ca; 4 Canadian Centre on Substance Use and Addiction, Ottawa, ON K1P 5E7, Canada; rgabrys@ccsa.ca; 5 Department of Psychiatry, University of Saskatchewan, Saskatoon, SK S7N 0W8, Canada; mansfieldmela@gmail.com

**Keywords:** mental health, substance use, concurrent experience, concurrent disorder, COVID-19, Canada

## Abstract

This study was aimed at identifying the prevalence of concurrent experience, poor mental health and problematic substance use, and its associated factors, among Canadian adults during the COVID-19 pandemic. A nationwide repeated cross-sectional sample of 14,897 Canadian adults (quota-sampled, weighted) were recruited on ten occasions between October 2020 and March 2022 using online panels. Concurrent experience was defined as mild to severe symptoms of depression (Patient Health Questionnaire-9) and/or anxiety (Generalized Anxiety Disorder-7) AND meeting screening criteria for problematic cannabis (Cannabis Use Disorder Identification Test-Revised) and/or problematic alcohol use (Alcohol Use Disorder Identification Test). Multivariable binary logistic regression models were fitted to identify the associated factors of concurrent experience using Stata v14.2 SE software. The pooled prevalence of concurrent experience was 17.12%, and 45.54% of the participants reported at least one experience (mental health symptoms or problematic substance use). The highest prevalence of concurrent experience per province was reported in Saskatchewan (19.4%) and the lowest in Quebec (13.6%). Younger adults, male respondents, those identifying as 2SLGBTQ+, self-reporting ethnocultural minority status, diagnostic history of mental health and substance use disorder, suicidal ideation, and lower ability to handle unexpected/difficult situations were significantly associated with concurrent experience during the COVID-19 pandemic in Canada. This analysis showed that the COVID-19 pandemic significantly impacted mental health and substance use in interrelated ways. Data-driven province-specific interventions might be helpful toward a client-centered and integrated mental health and substance use care system in Canada.

## 1. Introduction

In December 2019, Coronavirus disease 2019 (COVID-19) was first identified in an outbreak in Wuhan, China. Within weeks, it spread globally, leading the World Health Organization (WHO) to declare it a pandemic on 11 March 2020 [1]. Beyond its immediate impact on physical health outcomes and on mortality, the COVID-19 pandemic profoundly affected mental health and substance use patterns [2].

It is evident that large-scale traumatic events/humanitarian crises exacerbate the burden of mental illness among the affected population [3]. Similarly, the COVID-19 pandemic significantly impacted people in Canada and across the world. Some consider that adverse mental health is an associated but hidden pandemic [4]. A systematic review published at the end of 2021 reported that the global prevalences of major depressive disorder and anxiety disorder increased by 27.6% and 25.6%, respectively, during the first year of the pandemic compared to before the pandemic [5]. Statistics Canada’s COVID-19 and Mental Health survey reported that about 25% of Canadians aged 18 and older screened positive for symptoms of depression, anxiety, or posttraumatic stress disorder (PTSD) in the spring of 2021 [6].

Much like the increase in poor mental health, evidence also suggests that exposure to traumatic events or disasters have a profound effect on drug and alcohol use. To contain the spread of COVID-19, federal and provincial governments introduced individual and community-based measures, for example, individual measures such as self-monitoring, isolation, and quarantine, as well as community-based measures such as government implemented lockdown, proof of vaccine policies, and work from home. Evidence has shown that control measures like self-isolation and social distancing increase the risk of experiencing anger, anxiety, depression, boredom, etc., which, in turn, may shift to an increased risk of drug use as a coping mechanism [7,8]. Statistics Canada reported that among Canadians who had previously consumed alcohol (before the pandemic), nearly one-quarter (24%) reported their consumption had increased; among those who had previously consumed cannabis, more than one-third (34%) said their consumption had increased during the pandemic [9].

The relationship between psychiatric and substance use disorders and COVID-19 is complex. Several risk factors, for example comorbidities, are associated with both these disorders and increase the risk of exposure to and complications arising from the COVID-19 infection [10]. For example, evidence shows those with substance use disorders are more likely to experience worse COVID-19 outcomes, including risks of hospitalization and mortality [11].

The factors associated with depression, anxiety, and problematic alcohol and cannabis use are studied separately as individual outcomes. Evidence is sparse on who experiences, and the population burden of experiencing, both poor mental health and problematic substance use at the same time, referred to as concurrent experience [12] or as Concurrent disorder/Dual diagnosis [13]. One study, however, did report concurrency of increased drinking and symptoms of anxiety and depression [14]. During the early stages of COVID-19 in Canada, Mougharbel et al. [14] reported that about 10% of participants experienced both increased drinking and anxiety, and about 2.5% participants experienced both increased drinking and depression. However, Mougharbel et al. did not use a standardized scale to measure alcohol use, did not include cannabis use, and only captured data during the first five months of the pandemic. Further, Mougharbel et al. did not analyze the risk factors associated with this concurrent experience. To address these gaps, this study aims to provide a more comprehensive analysis of the concurrent experience of poor mental health and problematic substance use among Canadian adults during the first two years of the COVID-19 pandemic. The findings could serve as a foundation for further research into the intersectionality of mental health and substance use, particularly within the context of a global health emergency.

## 2. Materials and Methods

### 2.1. Data Source

We used data from a nationally representative repeated survey titled, ‘*The Relationship Between Mental Health and Substance Use During COVID-19*’, jointly commissioned by the Mental Health Commission of Canada (MHCC) and the Canadian Centre on Substance Use and Addiction (CCSA) and conducted in partnership with Leger (polling firm) [2]. Bimonthly cross-sectional surveys were conducted, commencing in October 2020 and closing in March 2022, across all provinces in Canada—a total of 10 cross-sectional surveys. A total of 16,797 eligible Canadian adults were included. Complete data from 14,897 Canadian adults were retained in the merged data set and analyzed in this study.

### 2.2. Poor Mental Health

#### 2.2.1. Depressive Symptoms

Depressive symptoms were assessed using the self-reported version of Patient Health questionnaire-9 (PHQ-9) [15]. PHQ-9 is based on nine diagnostic criteria of depression adapted from the Diagnostic and Statistical Manual of Mental Disorders, 4th ed. (DSM-IV) [15]. The PHQ-9 is widely used and validated for screening, aiding in diagnosis, and tracking the symptoms of depression and treatment progress among different population groups worldwide, including in Canada [16,17]. PHQ-9 asked for respondents’ experiences in tasks related to daily living during the past two weeks, for example—‘Little interest or pleasure in doing things’, ‘Feeling down, depressed or hopeless’, ‘Trouble falling asleep, staying asleep, or sleeping too much’, etc. Each item is evaluated on a severity scale ranging from 0 to 3 (0—not at all; 1—several days; 2—more than half of the days; 3—nearly every day), yielding a total score ranging 0—27, classified as follows: 0–9 none/mild depression, 10–19 moderate depression, and 20–27 severe depression [15]. The Cronbach’s alpha for the PHQ-9 scale was 0.918.

#### 2.2.2. Anxiety Symptoms

Anxiety symptoms were assessed using self-reported version of the Generalized Anxiety Disorder-7 (GAD-7) scale [18]. GAD-7 captured respondents experience of negative feelings, thoughts during the past two weeks, for example—‘Feeling nervous, anxious or on edge’, ‘Not being able to stop or control worrying’, ‘Trouble relaxing’, ‘Becoming easily annoyed or irritable’, etc. Each item of the GAD-7 scale is scored on a four-point (0–3) Likert scale, where 0—no difficulty at all and 3—extreme difficulty, with a total possible score of 21, classified as follows: 0–9 none/mild anxiety, 10–14 moderate anxiety, and 15–21 severe anxiety [18]. The Cronbach’s alpha for the GAD-7 scale was 0.930.

#### 2.2.3. Poor Mental Health

Population level studies have shown that those with a score of 10 or higher in PHQ-9/GAD-7, corresponding to moderate and severe symptoms, are suggested as having a clinically significant depression/anxiety [19,20]. Given this, a dichotomous variable was generated to identify poor mental health conditions (depression and anxiety). Participants having a PHQ-9 AND/OR GAD-7 score 10+ were categorized as having poor mental health (coded as Yes = 1, No = 0) [21].

### 2.3. Problematic Substance Use

#### 2.3.1. Problematic Alcohol Use

A modified version of the Alcohol Use Disorder Identification Test (AUDIT) scale was used to measure problematic alcohol use [22]. To better capture potential changes in problematic alcohol use during the pandemic, this modified version of the AUDIT scale used a reference period of the past six months instead of the past year. Ten different questions were asked regarding respondents’ alcohol use in the past six months, for example—‘How often do you have a drink containing alcohol?’, ‘How often do you have six or more drinks on one occasion?’, ‘Has a relative, friend, doctor, or other health care worker been concerned about your drinking or suggested you cut down?’, etc. The description of the response categories to each question varied, but the responses were given on a five-point Likert scale (0–4). The total was scored out of 40 and then categorized as 0–7 mild, 8–15 moderate, and 16+ severe [22]. The Cronbach’s alpha for the AUDIT scale was 0.875.

#### 2.3.2. Problematic Cannabis Use

The revised eight-item Cannabis Use Disorder Identification Test (CUDIT-R) scale was used to measure problematic or harmful cannabis use [23]. Participants were asked to respond to eight different question regarding their cannabis use in past six months, for example—‘How often do you use cannabis?’, ‘How often during the past six months did you fail to do what you was normally expected from you because of using cannabis?’, ‘How often do you use cannabis in situations that could be physically hazardous, such as driving, operating machinery, or caring for children?’, etc. Response options were set on five-point Likert scale (0–4), and the total score ranged between 0 to 32. The total score was then categorized as 0–7 mild, 8–11 moderate, and 12+ for severe problematic cannabis use [23]. The Cronbach’s alpha for the CUDIT-R scale was 0.832.

#### 2.3.3. Problematic Substance Use

A total score of 8 or higher on AUDIT or CUDIT-R, corresponding to moderate and high-risk use, was set as the cut-off to dichotomize alcohol or cannabis use. These thresholds have been previously shown to have sufficient sensitivity and specificity in screening for problematic use [24]. Therefore, in this study, participants having an AUDIT AND/OR CUDIT-R score 8+ were categorized as having problematic substance use (Yes = 1, No = 0) [25].

### 2.4. Outcome Variable: Concurrent Experience

The outcome variable of this study is ‘concurrent experience of poor mental health symptoms (depression and/or anxiety) and problematic substance use’. Participants having both poor mental health AND problematic substance use were categorized as having ‘concurrent experience’ (dichotomized as Yes = 1, No = 0).

### 2.5. Covariables

Dahlgren and Whitehead’s framework on the Social Determinants of Health was primarily followed for selecting the variables in this study, which was further justified by the existing literature [26]. The covariables considered in this study were as follows: (i) Age in years (categorized as 16–24, 25–34, 35–44, 45–54, 55–64, 65+); (ii) Gender identity (categorized as female, male, other); (iii) 2SLGBTQ+ identity (categorized as no, yes, prefer not to answer); (iv) Educational status (categorized as high school/less, college/diploma, university, don’t know/prefer not to answer); (v) Employment status (categorized as employed, unemployed, retired/student/unable to work, don’t know/prefer not to answer); (vi) Yearly household income-CAD (categorized as ≤20 K, 21 K–50 K, 51 K–100 K, >100 K); (vii) Number of family members in household (categorized as 2, 3–5, 6+); (viii) Migration status (categorized as Canadian-born, immigrant); (ix) Ethnocultural minority status (categorized as no, yes, prefer not to say); (x) Ever diagnosed with a mental health problem (categorized as no, yes); (xi) Ever diagnosed with a substance use disorder (no, yes); (xii) Ever experienced suicide ideation (no, yes); (xiii) Suicide ideation since the pandemic (no, yes); (xiv) Ability to handle unexpected/difficult situation (categorized as excellent/very good, good, fair/poor); and (xv) Smoking (no, yes).

### 2.6. Statistical Analysis

Both descriptive and inferential statistical tests were performed. Descriptive analysis (frequency, percentage) was performed to report the prevalence of the outcome variable (concurrent experience). A Pearson’s chi-square test was performed to test for distribution of prevalence of concurrent experience over the study variables. Crude and adjusted regression models were fitted to identify the factors associated with concurrent experience, and the results were presented as odd ratios (OR) and their 95% confidence intervals. Variance Inflation Factors (VIFs) were used to check the multicollinearity among the independent variables. A complete case analysis was performed during regression modeling. Interaction between a priori selected sociodemographic variables were used to check whether the associations with the outcome variable were modified (buffered or exacerbated) by equity-seeking groups; however, none of the interactions were significant, so it was not included in the final regression model. The data were analyzed using Stata (v14.2) software (StataCorp, College Station, TX, USA) using sampling weights calculated using the 2016 census). To handle missing data, a case-wise deletion method was followed. All associations were considered significant at a 5% level of significance.

## 3. Results

*Characteristics of study participants:* About one in five participants were aged 65 years and higher, and 51.3% of the survey population were female. The majority of participants had a university degree (46%), were employed (52.8%), and had a yearly household income between 51 K–100 K (CAD) (32%). About 19% of the participants were immigrants into Canada, and 21% self-identified as an ethnocultural minority. About 30% of the participants had a clinical history of mental health problems. About 39% of participants reported that they had an excellent/very good ability to handle unexpected/difficult situations, and 26.3% reported fair/poor ability (Table 1).

**Figure 1:**
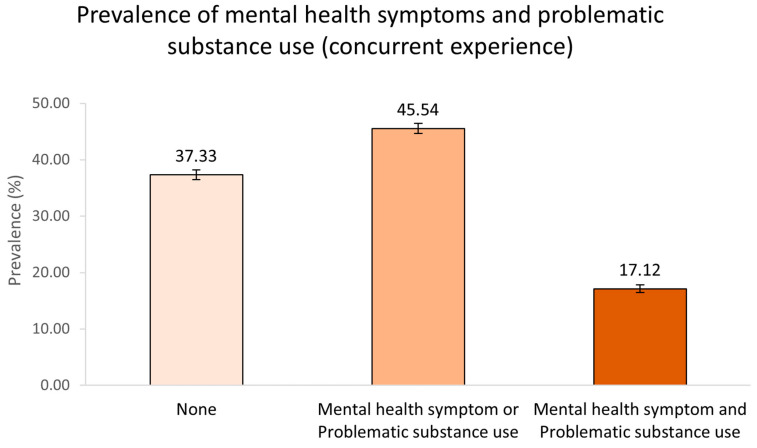
Prevalence of ‘concurrent experience’ (anxiety and/or depression symptoms and problematic alcohol and/or cannabis use) among Canadian adults, October 2020–March 2022 (note: error bars indicate 95% CI).

**Figure 2:**
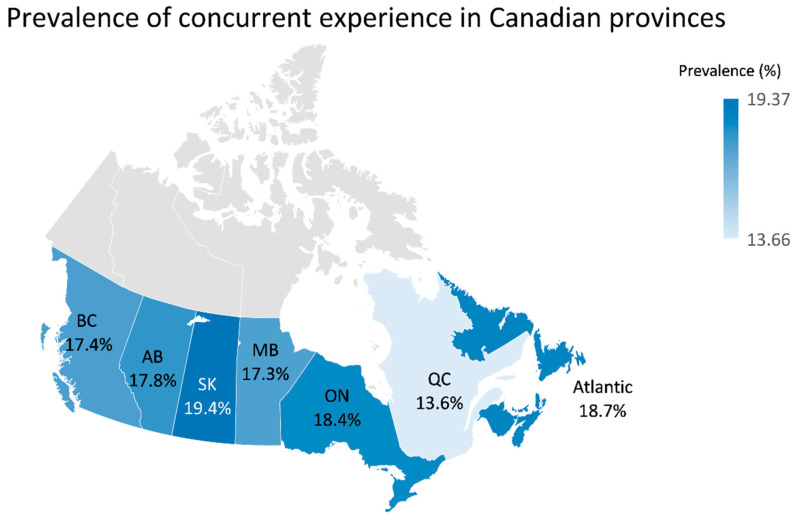
Prevalence map of ‘concurrent experience’ (anxiety and/or depression symptoms and problematic alcohol and/or cannabis use) across Canadian provinces.

*Prevalence of concurrent experience:* Figure 1 depicts that the pooled prevalence of concurrent experience (poor mental health AND problematic substance use) was 17.12% (95% CI: 16.44% to 17.81%) among respondents during the COVID-19 pandemic. As shown in Figure 2, the highest prevalence of concurrent experience was reported in Saskatchewan (19.4%) and the lowest prevalence was reported in Quebec (13.6%). About 18.4% of the participants living in Ontario (the most populated province in Canada) experienced concurrent experience of poor mental health and problematic substance use during the COVID-19 pandemic. The prevalence of concurrent experience according to the characteristics of the study participants are presented in Supplementary Table S1.

*Factors associated with concurrent experience:* The factors associated with concurrent experience of poor mental AND problematic substance use (both crude and adjusted model) are presented in Table 2. The adjusted logistic model suggests that younger participants had higher odds for concurrent experience compared to those who were 65 years or older. Males were 62% more likely to report concurrent experience compared to female (aOR = 1.62, 95% CI: 1.39 to 1.89). Those who were retired/student/unable to work were 21% less likely to report concurrent experience compared to those who were employed (aOR = 0.79, 95% CI: 0.64 to 0.97). A history of mental health problems, substance use disorder, and suicidal ideation were also significant correlates of concurrent experience. Those who experienced suicidal ideation since the pandemic were 46% more likely to report concurrent experience (aOR = 1.46, 95% CI: 1.11 to 1.91). Participants’ ability to handle unexpected/difficult situations was negatively associated with concurrent experience. Those who stated that they had fair/poor ability were 226% more likely to report concurrent experience than excellent/very good (aOR = 2.26, 95% CI: 1.84 to 2.76). Those who indicated that they were smokers were almost 400% more likely to have concurrent experience compared to non-smokers (aOR = 3.96, 95% CI: 3.38 to 4.63). The province-specific risk factors calculation suggests that the risk factors for concurrent experience were not constant and varied from province to province. There were some common risk factors and some unique for each province, which is presented in Supplementary Table S2.

## 4. Discussion

This study aimed to estimate the prevalence and associated factors of concurrent experience (poor mental health and problematic substance use) among a sample of Canadian adults during the first two years of the COVID-19 pandemic. The pooled prevalence of concurrent experience was 17.12%, and, among the Canadian provinces, the highest prevalence of concurrent experience was found in Saskatchewan and the lowest in Quebec. The age of the participants, sex, employment status, diagnostic history of mental health problems and substance use disorder, suicidal ideation, ability to handle unexpected/difficult situations, and smoking behavior were significant risk factors for concurrent experience of poor mental health and problematic substance use during the pandemic.

The pooled prevalence of concurrent experience found in this survey was substantially higher than a previous study where the prevalence of drinking and anxiety was about 10% and the prevalence of drinking and depression was 2.5% among Canadian adults [14]. The reason behind the difference in the prevalence of concurrent experience between the two studies may be due to different data collection timelines and different operational definitions for the outcome variable. Mougharbel et al. used data that were collected just after two months of the declaration of the COVID-19 as a global pandemic [14]. Since the spread of COVID-19 was not consistent, it is important to track/monitor for a longer period to better explain the impact of the pandemic on mental health and substance use. While most previous studies looked at the impact of the pandemic on Canadians’ either mental health or substance use, the findings of this research could not be compared with other studies in Canada.

In terms of the risk factors associated with concurrent experience, the odds of concurrent experience were found to be significantly higher among the younger age group compared to the older adults (65+ years of age), which is aligned with the previous study [14]. The highest odds of experiencing concurrent experience were found among the 16–24 years age group. This age group mainly represents young people in post-secondary educational institutions, who may have fewer coping resources as alternatives to using alcohol and/or cannabis. In addition, young people are more reliant on social interactions for identity development and emotional support. They have a different social life than older individuals. Activities such as sports, hobbies, and social events play a crucial role in adding structure to their day and help with coping and stress relief for young people. The disruptions to such activities, therefore, limited healthy outlets for stress and energy, potentially leading to an increased risk of concurrent experience. Similarly, the odds of reporting concurrent experience decreased with age. This may be due to the fact that older adults have more coping ability and a higher capacity to adapt, endure, and bounce back during a time of crisis due to their accumulated life experience and financial stability [27].

This analysis showed that males were more likely to experience concurrent experience compared with females. Interestingly, a previous study found that those who were female were associated with higher odds of reporting increased alcohol consumption and anxiety [14]. In a separate analysis (not presented in this study), we found that females were more likely to report poor mental health conditions, while males were more likely to report problematic substance use. However, when we bring these two experiences together, those experiencing more severe, or acute, negative experiences—males—seem to be at higher risk compared to females. This finding may reflect gender-specific coping mechanisms, where males are more prone to externalize behaviors, such as substance use, while females may internalize stress, leading to mental health challenges. However, studies directly addressing the intersection of these concurrent experiences are limited, highlighting the need for further research to understand the underlying mechanism. Participants who were retired/students/unable to work showed a protective factor for concurrent experience of poor mental health and problematic substance use. This can be explained by the fact that these population groups likely did not have any work-related stress or fear of losing their job; they may have enough time for self-care or received care and social support from their family and friends.

A diagnosed history of mental health problems and substance use disorder was also a risk factor for concurrent experience. A systematic review reported that people with previous mental health problems had a significantly higher prevalence of poor mental health symptoms during the COVID-19 pandemic (i.e., depression, anxiety) [28]. Individuals with pre-existing mental health conditions, who may already have struggled with feelings of loneliness or difficulty forming and maintaining relationships, were socially isolated due to the pandemic and may have experienced worsened mental health conditions. In addition to this, the pandemic strained mental health support systems, including limited access to in-person therapy sessions and reduced availability of community resources. Individuals with previous mental health problems may have experienced challenges in receiving the necessary support and treatment they relied upon before the pandemic. Limited access to care was made worse in subgroups of people with mental health difficulties where virtual care was unavailable among those with low socioeconomic status and when it elicited suspiciousness in those with paranoia [29,30]. This lack of support may have further contributed to the deterioration of their mental health [31]. Evidence also suggests that Canadians who have pre-existing poor mental health status were more likely to consume more alcohol during the pandemic [32].

Suicidal ideation was another risk factor for concurrent experience. A study conducted at the end of the first year of COVID-19 pandemic among Canadian adults reported that participants with depression and anxiety symptoms, and who increased their consumption of cannabis, were at significantly higher risk of suicidal ideation [33,34]. Participants’ ability to handle unexpected/difficult situations was also significantly associated with concurrent experience. Individuals who possess good abilities to handle an unexpected situation often have a higher coping capacity, so they can navigate the situation more effectively and manage their emotional and psychological well-being better. Lastly, smoking was identified as a risk factor for concurrent experience in this study. A previous study also reported that there is a causal relationship between smoking and mental health symptoms [35], and smoking status increases the risk of alcohol misuse [36]. Nicotine found in cigarettes affects the release of chemicals in the brain, such as dopamine and serotonin, leading to temporary mood elevation and relaxation. However, when these effects wear off, smokers may experience mood swings, depression, or anxiety [37]. It is also reported that smoking often co-occurs with other substance use, such as alcohol or drugs, which can further worsen mental health conditions [38].

Province-specific analysis showed that the risk factors for experiencing concurrent experience were not the same for all provinces; this may be due to the fact that people living in different provinces of Canada have different lifestyles and social contexts; in addition, people’s reaction to the government policy to contain the spread of COVID-19 was not the same, for example, we have seen anti-mask protests in different cities of Canada during the pandemic. This analysis highlighted the importance of data-driven province-specific interventions to build a client-centered and integrated mental health and substance use care system in Canada.

We acknowledge several limitations of our study. First, due to the cross-sectional nature of the survey, inferences of causality in relation to the risk factors associated with concurrent experience cannot be made. Second, that the participants were selected from an existing web panel and did not include participants from the Territories in Canada limits the generalizability of the study findings to the Territories. Third, a non-random quota sampling technique was followed for data collection; therefore, the survey results are not truly representative of all Canadian adults. Due to the self-reported nature of the data, there are possibilities of recall and/or social desirability biases which may have an influence on these findings. Since we have used data from serial cross-sectional surveys, there is a possibility of double counting some individuals who may have participated in more than one wave of surveying, which might also impact the findings of this analysis. This could introduce bias into the analysis by over-representing certain responses and skewing the overall findings. Last but not least, this study failed to control for (due to the lack of available data) factors like personality traits, which are known risk factors for both mental health and substance use [39,40]. We suggest caution while interpreting the findings of this study.

## 5. Conclusions

This study sheds light on the unexplored area of concurrent experience of poor mental health and problematic substance use by Canadian adults during the period of the COVID-19 pandemic. This research found that one in six Canadian adults reported concurrent poor mental health and problematic substance use—a prevalence that is higher than we had expected and had been previously reported. Younger people, males, those who had a history of mental health and/or substance use problems, smokers, and those who said that they were less able to handle unexpected or difficult situations were more likely to indicate concurrent poor mental health and problematic substance use. Those who are retired from work, students, or unable to work are less likely to have concurrent experience. This research has underscored the importance of holistic and integrated treatment models that simultaneously address mental health and substance use disorders. The findings of our research strongly support the implementation of integrated interventions that incorporate psychotherapy, pharmacotherapy, and social support to provide comprehensive care tailored to the specific needs of individuals with concurrent experience. We expect our research will serve as a catalyst for further studies and encourage a meaningful change in policy and practice, ultimately leading to better support and treatment for individuals with concurrent experience of poor mental health and problematic substance use in Canada.

## Figures and Tables

**Table 1:** Characteristics of the study participants of the survey entitled *Leger Pool: The Relationship Between Mental Health and Substance Use During COVID-19*; data collected during October 2020 to March 2022 in Canada.

Characteristics	Frequency (n)	Percent (%)
**Age (years)**		
16–24	2254	13.42
25–34	2681	15.96
35–44	2639	15.71
45–54	2925	17.41
55–64	2851	16.97
65+	3447	20.52
**Gender**		
Female	8625	51.35
Male	7964	47.41
Others	208	1.24
**2SLGBTQ+ identity**		
No	14,776	87.97
Yes	1854	11.03
Prefer not to answer	167	0.99
**Educational status**		
High school/less	3726	22.18
College/diploma	5231	31.14
University	7711	45.90
Don’t know/prefer not to answer	129	0.77
**Employment**		
Employed	8865	52.78
Unemployed	1331	7.92
Retired/student/unable to work	6379	37.98
Don’t know/prefer not to answer	222	1.32
**Yearly household income (CAD)**		
≤20 K	1353	8.05
21 K–50 K	3903	23.24
51 K–100 K	5391	32.09
>100 K	4323	25.74
Missing	1827	10.88
**No. of family members**		
2	6391	38.05
3–5	5779	34.40
6+	466	2.78
Prefer not to answer	188	1.12
Missing	3974	23.66
**Migration status**		
Canadian born	13,405	79.81
Immigrant	3247	19.33
Don’t know/prefer not to answer	145	0.86
**Ethnocultural minor (self-identified)**		
No	12,974	77.24
Yes	3563	21.21
Prefer not to answer	260	1.55
**Ever diagnosed with mental health problem**		
No	11,344	67.54
Yes	5068	30.17
Don’t know/prefer not to answer	385	2.30
**Ever diagnosed with substance use disorder**		
No	15,808	94.11
Yes	805	4.80
Don’t know/prefer not to answer	184	1.10
**Ever suicide ideation**		
No	13,189	78.52
Yes	2850	16.96
Don’t know/prefer not to answer	758	4.52
**Suicide ideation-since pandemic**		
No	14,975	89.15
Yes	1261	7.51
Don’t know/prefer not to answer	561	3.35
**Ability to handle unexpected/difficult situation**		
Excellent/very good	6549	38.99
Good	5568	33.15
Fair/poor	4414	26.28
Missing	266	1.59
**Smoking**		
No	11,257	67.02
Yes	2864	17.05
Prefer not to say	175	1.04
Missing	2502	14.90

Note: Weighted frequency and percentages are reported after rounding.

**Table 2:** Factors associated with concurrent experience (poor mental health AND problematic substance use).

Characteristics	Crude Model, OR [95% CI]	Adjusted Model †, OR [95% CI]
**Age (years)**		
16–24	5.80 [4.66–7.21] ***	3.37 [2.36–4.80] ***
25–34	7.08 [5.78–8.68] ***	3.28 [2.33–4.61] ***
35–44	4.85 [3.95–5.95] ***	2.29 [1.62–3.22] ***
45–54	3.20 [2.59–3.96] ***	1.79 [1.28–2.51] **
55–64	2.03 [1.61–2.55] ***	1.38 [1.00–1.92] *
65+	Reference	Reference
**Gender**		
Female	Reference	Reference
Male	1.57 [1.42–1.73] ***	1.62 [1.39–1.89] ***
**2SLGBTQ+ identity**		
No	Reference	Reference
Yes	2.43 [2.13–2.76] ***	1.11 [0.89–1.38]
**Educational status**		
High school/less	1.02 [0.90–1.15]	0.93 [0.75–1.14]
College/diploma	0.96 [0.85–1.07]	1.07 [0.90–1.28]
University	Reference	Reference
**Employment**		
Employed	Reference	Reference
Unemployed	1.74 [1.49–2.03] ***	1.12 [0.87–1.45]
Retired/student/unable to work	0.53 [0.47–0.60] ***	0.79 [0.64–0.97] *
**Yearly household income (CAD)**		
≤20 K	1.77 [1.48–2.12] ***	0.99 [0.71–1.38]
21 K–50 K	1.49 [1.30–1.70] ***	0.92 [0.75–1.14]
51 K–100 K	1.15 [1.00–1.31] *	0.87 [0.73–1.04]
>100 K	Reference	Reference
**No. of family members**		
2	Reference	Reference
3–5	1.38 [1.22–1.55] ***	0.91 [0.78–1.07]
6+	1.52 [1.11–2.08] **	0.79 [0.51–1.20]
**Migration status**		
Canadian born	Reference	Reference
Immigrant	1.12 [0.99–1.26]	0.88 [0.73–1.07]
**Ethnocultural minor (self-reported)**		
No	Reference	Reference
Yes	1.39 [1.24–1.55] ***	0.86 [0.71–1.03]
**Ever diagnosed with mental health problem**		
No	Reference	Reference
Yes	2.80 [2.54–3.10] ***	1.63 [1.39–1.92] ***
**Ever diagnosed with substance use disorder**		
No	Reference	Reference
Yes	9.28 [7.80–11.03] ***	3.49 [2.62–4.65] ***
**Ever suicide ideation**		
No	Reference	Reference
Yes	3.27 [2.93–3.66] ***	1.35 [1.09–1.68] **
**Suicide ideation-since pandemic**		
No	Reference	Reference
Yes	4.33 [3.75–5.00] ***	1.46 [1.11–1.91] **
**Ability to handle unexpected/difficult situation**		
Excellent/very good	Reference	Reference
Good	1.61 [1.42–1.82] ***	1.92 [1.60–2.32] ***
Fair/poor	2.72 [2.41–3.08] ***	2.26 [1.84–2.76] ***
**Smoking**		
Yes	5.50 [4.92–6.14] ***	3.96 [3.38–4.63] ***
No	Reference	Reference

* *p* < 0.05, ** *p* < 0.01, *** *p* < 0.001. † Adjusted with all the variables in the table in addition to data collection timeline and province. Model fit statistics (Hosmer–Lemeshow test): F = 1.23, *p* = 0.2726.

## Data Availability

The data used in this study are owned by MHCC and CCSA and shared with Dr Nazeem Muhajarine (NM) under a data sharing agreement. The data sets generated and/or analyzed during the current study are available from NM on reasonable request.

## Supplementary Materials

The following supporting information can be downloaded at: https://www.mdpi.com/article/10.3390/ijerph21121644/s1. Table S1: Prevalence of concurrent experience according to the study variables and measures of association with the Cho-square test. Table S2: Factors associated with concurrent experience in different provinces of Canada.
