# Supplementary material for: Concurrent Experience of Self-Reported Mental Health Symptoms and Problematic Substance Use During the First Two Years of the COVID-19 Pandemic Among Canadian Adults: Evidence from a Repeated Nationwide Cross-Sectional Survey
Source: Int J Environ Res Public Health. 2024 Dec 10;21(12):1644. doi: 10.3390/ijerph21121644 (PMC11675694; doi:10.3390/ijerph21121644)
Supplement: Supplementary file 1 [file ijerph-21-01644-s001.zip › ijerph-3229821-supplementary.pdf]

Supplement table 1. Prevalence of dual experience according to the study variables and measures of association with the Chi-square test.

Characteristics		Dual experience (%)	95% CI	p-value
Age (years)				
	16–24	25.00	22.70–27.45	<0.001
	25–34	28.93	26.95–31.00	
	35–44	21.79	20.08–23.61	
	45–54	15.56	14.11–17.12	
	55–64	10.45	9.16–11.90	
	65+	5.43	4.59–6.42	
Sex				
	Female	13.92	13.10–14.78	<0.001
	Male	20.30	19.23–21.41	
2SLGBTQ+ identity				
	No	15.29	14.61–16.00	<0.001
	Yes	30.51	28.08–33.06	
Educational status				
	High school/ less	17.50	16.09–19.02	0.546
	College/ diploma	16.64	15.45–17.89	
	University	17.19	16.20–18.23	
Employment				
	Employed	19.40	18.44–20.39	<0.001
	Unemployed	29.53	26.70–32.54	
	Retired/ student/ unable to work	11.44	10.50–12.44	
Yearly household income (CAD)				
	≤ 20K	24.07	21.47–26.88	<0.001
	21K–50K	21.02	19.52–22.60	
	51K–100K	17.02	15.85–18.27	
	> 100K	15.13	13.90–16.45	
No. of family members				
	2	13.25	12.29–14.27	<0.001
	3–5	17.42	16.27–18.63	
	6+	18.87	14.71–23.89	
Migration status				
	Canadian born	16.83	16.08–17.60	0.118
	Immigrant	18.49	16.91–20.17	
Ethnocultural minor (self-reported)				
	No	16.12	15.38–16.90	<0.001
	Yes	21.13	19.56–22.80	
Ever diagnosed with mental health problem				
	No	12.08	11.37–12.82	<0.001
	Yes	27.83	26.39–29.32	
Ever diagnosed with substance use disorder				
	No	14.49	13.84–15.16	<0.001
	Yes	61.13	57.16–64.95	
Ever suicide ideation				
	No	12.88	12.21–13.59	<0.001
	Yes	32.65	30.65–34.73	
Suicide ideation- since pandemic				
	No	14.10	13.44–14.78	<0.001

	Yes	41.57	38.41–44.80	
Ability to handle unexpected/difficult situation				
	Excellent/ very good	11.28	10.38–12.25	<0.001
	Good	17.02	15.86–18.25	
	Fair/ poor	25.76	24.27–27.31	
Smoking				
	Yes	38.03	36.02–40.07	<0.001
	No	10.03	9.42–10.68	

Supplement table 2. Factors associated with concurrent experience in different provinces of Canada.

**Characteristics**	**ON**	**QC**	**MB**
	**AOR† [95% CI]**	**AOR† [95% CI]**	**AOR† [95% CI]**
Age (years)			
16–24	3.26 [1.77–6.00] ***	2.91 [1.34–6.30] **	3.98 [1.17–13.52] *
25–34	3.69 [2.14–6.36] ***	2.40 [1.06–5.41] *	4.19 [1.19–14.66] *
35–44	2.40 [1.40–4.13] **	2.14 [0.96–4.77]	2.40 [0.66–8.72]
45–54	2.00 [1.16–3.44] *	1.43 [0.65–3.11]	1.32 [0.34–5.08]
55–64	1.71 [1.01–2.89] *	0.98 [0.44–2.20]	0.12 [0.02–0.66] *
65+	Ref.	Ref.	Ref.
Gender			
Female	Ref.	Ref.	Ref.
Male	2.02 [1.58–2.59] ***	1.39 [1.00–1.94] *	1.84 [0.98–3.46]
2SLGBTQ+ identity			
No	Ref.	Ref.	Ref.
Yes	1.27 [0.89–1.80]	0.86 [0.53–1.38]	1.33 [0.60–2.95]
Educational status			
High school/ less	0.74 [0.52–1.06]	1.28 [0.84–1.94]	0.97 [0.41–2.31]
College/ diploma	0.96 [0.73–1.28]	1.16 [0.79–1.70]	0.54 [0.25–1.14]
University	Ref.	Ref.	Ref.
Employment			
Employed	Ref.	Ref.	Ref.
Unemployed	1.36 [0.89–2.06]	1.32 [0.70–2.49]	0.71 [0.23–2.18]
Retired/ student/ unable to work	0.79 [0.56–1.10]	0.69 [0.43–1.10]	0.78 [0.32–1.89]
Yearly household income			
≤ 20K CAD	0.81 [0.45–1.47]	1.16 [0.59–2.26]	1.09 [0.25–4.67]
21K–50K CAD	0.86 [0.61–1.23]	0.93 [0.60–1.43]	0.88 [0.31–2.48]
51K–100K CAD	0.99 [0.74–1.30]	0.74 [0.51–1.08]	1.21 [0.57–2.52]
> 100K CAD	Ref.	Ref.	Ref.
No. of family members			
2	Ref.	Ref.	Ref.
3–5	0.98 [0.75–1.28]	0.86 [0.61–1.22]	0.47 [0.24–0.93] *
6+	0.97 [0.54–1.72]	1.12 [0.45–2.81]	0.12 [0.02–0.65] *
Migration status			
Canadian born	Ref.	Ref.	Ref.
Immigrant	0.80 [0.59–1.08]	1.10 [0.68–1.77]	0.85 [0.40–1.81]
Ethnocultural minor (self-reported)			
No	Ref.	Ref.	Ref.
Yes	0.82 [0.62–1.10]	1.12 [0.70–1.81]	1.01 [0.47–2.17]
Ever diagnosed with mental health problem			
No	Ref.	Ref.	Ref.
Yes	1.94 [1.47–2.55] ***	1.45 [1.03–2.04] *	2.59 [1.35–5.00] **
Ever diagnosed with substance use disorder			
No	Ref.	Ref.	Ref.
Yes	2.72 [1.67–4.44] ***	10.20 [5.07–20.51] ***	2.69 [0.96–7.49]
Ever suicide ideation			
No	Ref.	Ref.	Ref.
Yes	1.15 [0.78–1.68]	1.61 [0.98–2.64]	2.69 [1.22–5.91] *
Suicide ideation- since pandemic			
No	Ref.	Ref.	Ref.
Yes	1.83 [1.16–2.88] **	0.71 [0.35–1.43]	0.72 [0.22–2.37]
Ability to handle unexpected/difficult situations			

Excellent/ very good	Ref.	Ref.	Ref.
Good	1.74 [1.29–2.34] ***	1.70 [1.13–2.55] *	1.56 [0.75–3.28]
Fair/ poor	1.70 [1.24–2.34] **	3.27 [2.08–5.23] ***	1.87 [0.86–4.05]
Smoking			
Yes	4.43 [3.42–5.74] ***	2.80 [1.97–3.98] ***	9.25 [4.81–17.80] ***
No	Ref.	Ref.	Ref.

Supplement table 2. Factors associated with concurrent experience in different provinces of Canada (contd.)

**Characteristics**	**SK**	**AB**	**BC**
	**AOR† [95% CI]**	**AOR† [95% CI]**	**AOR† [95% CI]**
Age (years)			
16–24	4.63 [1.28–16.78] *	2.26 [0.71–7.17]	6.34 [2.37–16.96] ***
25–34	4.69 [1.35–16.23] *	4.37 [1.46–13.00] **	3.21 [1.23–8.39] *
35–44	1.56 [0.42–5.79]	2.34 [0.75–7.30]	3.13 [1.18–8.34] *
45–54	1.91 [0.54–6.74]	1.63 [0.49–5.37]	2.49 [0.98–6.30]
55–64	1.37 [0.37–5.10]	1.26 [0.42–3.81]	1.81 [0.71–4.57]
65+	Ref.	Ref.	Ref.
Gender			
Female	Ref.	Ref.	Ref.
Male	1.65 [0.94–2.88]	1.28 [0.79–2.06]	1.26 [0.81–1.97]
2SLGBTQ+ identity			
No	Ref.	Ref.	Ref.
Yes	1.31 [0.52–3.32]	1.16 [0.50–2.68]	1.04 [0.54–1.97]
Educational status			
High school/ less	0.40 [0.19–0.83] *	1.07 [0.56–2.02]	1.22 [0.66–2.25]
College/ diploma	0.59 [0.32–1.09]	1.98 [1.19–3.32] **	1.26 [0.77–2.06]
University	Ref.	Ref.	Ref.
Employment			
Employed	Ref.	Ref.	Ref.
Unemployed	1.31 [0.54–3.21]	0.58 [0.28–1.21]	1.39 [0.67–2.86]
Retired/ student/ unable to work	0.40 [0.17–0.93] *	0.68 [0.36–1.26]	1.16 [0.66–2.03]
Yearly household income			
≤ 20K CAD	8.63 [3.19–23.30] ***	0.71 [0.30–1.68]	0.33 [0.08–1.39]
21K–50K CAD	1.01 [0.47–2.17]	0.79 [0.38–1.61]	0.81 [0.45–1.46]
51K–100K CAD	1.25 [0.65–2.38]	0.78 [0.45–1.34]	0.70 [0.42–1.18]
> 100K CAD	Ref.	Ref.	Ref.
No. of family members			
2	Ref.	Ref.	Ref.
3–5	1.12 [0.65–1.92]	1.32 [0.79–2.22]	0.97 [0.59–1.58]
6+	0.50 [0.07–3.56]	0.60 [0.19–1.88]	1.00 [0.28–3.57]
Migration status			
Canadian born	Ref.	Ref.	Ref.
Immigrant	0.47 [0.19–1.12]	0.90 [0.48–1.69]	1.22 [0.70–2.10]
Ethnocultural minor (self-reported)			
No	Ref.	Ref.	Ref.
Yes	0.75 [0.37–1.54]	1.01 [0.59–1.72]	0.52 [0.30–0.88] *
Ever diagnosed with mental health problem			
No	Ref.	Ref.	Ref.
Yes	1.05 [0.59–1.87]	1.87 [1.14–3.06] *	1.37 [0.81–2.31]
Ever diagnosed with substance use disorder			
No	Ref.	Ref.	Ref.

Yes	3.08 [1.28–7.43] *	2.49 [1.08–5.72] *	3.86 [1.62–9.15] **
Ever suicide ideation			
No	Ref.	Ref.	Ref.
Yes	2.17 [1.03–4.58] *	1.44 [0.79–2.65]	1.34 [0.72–2.49]
Suicide ideation- since pandemic			
No	Ref.	Ref.	Ref.
Yes	1.67 [0.62–4.45]	1.36 [0.61–3.03]	2.19 [1.03–4.62] *
Ability to handle unexpected/difficult situations			
Excellent/ very good	Ref.	Ref.	Ref.
Good	1.02 [0.52–2.02]	3.44 [1.75–6.76] ***	2.70 [1.56–4.65] ***
Fair/ poor	1.57 [0.79–3.11]	3.60 [1.79–7.24] ***	2.92 [1.56–5.46] **
Smoking			
Yes	4.27 [2.41–7.54] ***	5.62 [3.33–9.46] ***	3.97 [2.47–6.37] ***
No	Ref.	Ref.	Ref.

Supplement table 2. Factors associated with concurrent experience in different provinces of Canada (contd.)

**Characteristics**	**AT**
	**AOR† [95% CI]**
Age (years)	
16–24	4.47 [1.34–14.87] *
25–34	5.89 [1.58–21.88] **
35–44	2.92 [0.83–10.28]
45–54	1.77 [0.56–5.63]
55–64	2.30 [0.85–6.23]
65+	Ref.
Gender	
Female	Ref.
Male	1.76 [0.96–3.24]
2SLGBTQ+ identity	
No	Ref.
Yes	2.01 [0.92–4.38]
Educational status	
High school/ less	0.76 [0.38–1.51]
College/ diploma	0.58 [0.30–1.11]
University	Ref.
Employment	
Employed	Ref.
Unemployed	1.06 [0.47–2.39]
Retired/ student/ unable to work	1.20 [0.60–2.40]
Yearly household income	
≤ 20K CAD	1.96 [0.74–5.15]
21K–50K CAD	1.40 [0.67–2.90]
51K–100K CAD	0.73 [0.35–1.52]
> 100K CAD	Ref.
No. of family members	
2	Ref.
3–5	0.47 [0.27–0.84] *
6+	0.24 [0.02–2.05]
Migration status	
Canadian born	Ref.
Immigrant	0.50 [0.21–1.18]
Ethnocultural minor (self-reported)	

No	Ref.
Yes	1.43 [0.68–2.99]
Ever diagnosed with mental health problem	
No	Ref.
Yes	1.18 [0.67–2.07]
Ever diagnosed with substance use disorder	
No	Ref.
Yes	6.19 [2.44–15.66] ***
Ever suicide ideation	
No	Ref.
Yes	1.32 [0.62–2.81]
Suicide ideation- since pandemic	
No	Ref.
Yes	1.71 [0.68–4.30]
Ability to handle unexpected/difficult situations	
Excellent/ very good	Ref.
Good	1.86 [0.89–3.88]
Fair/ poor	2.50 [1.17–5.32] *
Smoking	
Yes	4.94 [2.80–8.71] ***
No	Ref.

* $p < 0.05$, ** $p < 0.005$, *** $p < 0.001$; †Adjusted with all the variables in the table

Note: AOR = Adjusted Odd Ratio, ON = Ontario, QC = Quebec, MB = Manitoba, SK = Saskatchewan, AB = Alberta, BC = British Columbia, and AT = Atlantic provinces.
